# Leveraging Time–Frequency Distribution Priors and Structure-Aware Adaptivity for Wideband Signal Detection and Recognition in Wireless Communications

**DOI:** 10.3390/s25247650

**Published:** 2025-12-17

**Authors:** Xikang Wang, Hua Xu, Zisen Qi, Qingwei Meng, Hongcheng Fan, Yunhao Shi, Wenran Le

**Affiliations:** Information and Navigation School, Air Force Engineering University of PLA, Xi’an 710077, China; wangxkmaki@163.com (X.W.); qizisen@163.com (Z.Q.); qingw_meng@163.com (Q.M.); hongcheng_fan@126.com (H.F.); shiyunhaoai@163.com (Y.S.); wenran070303@163.com (W.L.)

**Keywords:** deep learning, time-frequency localization, wideband signal detection and recognition, post-processing

## Abstract

Wideband signal detection and recognition (WSDR) is considered an effective technical means for monitoring and analyzing spectra. The mainstream technical route involves constructing time–frequency representations for wideband sampled signals and then achieving signal detection and recognition through deep learning-based object detection models. However, existing methods exhibit insufficient attention on the prior information contained in the time–frequency domain and the structural features of signals, leaving ample room for further exploration and optimization. In this paper, we propose a novel model called TFDP-SANet for the WSDR task, which is based on time–frequency distribution priors and structure-aware adaptivity. Initially, considering the horizontal directionality and banded structure characteristics of the signal in the time–frequency representation, we introduce both the Strip Pooling Module (SPM) and Coordinate Attention (CA) mechanism during the feature extraction and fusion stages. These components enable the model to aggregate long-distance dependencies along horizontal and vertical directions, mitigate noise interference outside local windows, and enhance focus on the spatial distributions and shape characteristics of signals. Furthermore, we adopt an adaptive elliptical Gaussian encoding strategy to generate heatmaps, which enhances the adaptability of the effective guidance region for center-point localization to the target shape. During inference, we design a Time–Frequency Clustering Optimizer (TFCO) that leverages prior information to adjust the class of predicted bounding boxes, further improving accuracy. We conduct a series of ablation experiments and comparative experiments on the WidebandSig53 (WBSig53) dataset, and the results demonstrate that our proposed method outperforms existing approaches on most metrics.

## 1. Introduction

With the rapid development of communication network ecosystems such as intelligent vehicular networks and industrial internet, the demand for high-speed and reliable communication services has seen substantial growth. This trend has made electromagnetic environments increasingly complex, thereby driving in-depth research into cognitive electromagnetic spectrum technologies. Particularly in non-cooperative communication scenarios, the integration of wideband sampling and intelligent spectrum sensing technology has emerged as a critical enabler for achieving cognitive electromagnetic spectrum capabilities. Its accuracy and reliability directly and profoundly impact subsequent tasks such as signal demodulation and decoding, spectrum occupancy analysis, and spectrum management. Within specific frequency bands, wideband signal detection and recognition (WSDR) is regarded as an effective technical means for spectrum monitoring and analysis, capable of realizing time–frequency localization and modulation classification of multiple signals. WSDR is flexibly applicable to both military and civilian fields, addressing the challenges posed by a large number of heterogeneous wireless signals in complex electromagnetic spaces, and thus has attracted widespread attention in areas such as the Internet of Things, intelligent communications, and electronic warfare.

Generally, there are two subtasks in WSDR, consisting of signal detection (SD) and modulation recognition (MR). SD can detect multiple signal objects in the wideband spectrum, and estimate signal parameters such as center frequency and time–frequency span. At the same time, MR assigns a modulation class to each signal object, which may be in the known class set or the unknown class. Traditional SD methods are mainly used to determine the presence of signals in the narrowband. Among them, the widely employed methods include matching filters [1], cyclostationary detection [2], energy detection [3], and eigenvalue detection [4]. However, these methods suffer from several critical limitations, including sensitivity to variations in the signal-to-noise ratio (SNR) and constraints on both the quantity of estimable parameters and estimation accuracy [5]. For the wideband spectrum, traditional SD methods need to be combined with channelization techniques such as tunable filters or filter banks [6], which put high demands on both computational complexity and filter design. Traditional MR methods mainly include likelihood-based methods and feature-based methods. The methods based on maximum likelihood treat the MR problem as a multivariate hypothesis testing problem, which can obtain optimal solutions, but their computational complexity is relatively high and they are difficult to apply in practice. Methods based on expert features rely heavily on domain-specific knowledge and manual parameter tuning, which limits their applicability and generalization capability across diverse scenarios. With the successful application of deep learning models in sequences and images, researchers have explored intelligent SD [7,8] and intelligent MR [9] from these two perspectives. In the traditional WSDR task, SD and MR are performed independently. The current trend is to use multitask joint optimization to carry out WSDR. It is typical to use an object detection model to detect and recognize signal objects in the time–frequency domain. Compared with separated detection and recognition methods, the integrated framework is more concise.

However, unlike general object detection tasks, signal objects in the time–frequency domain often exhibit extreme aspect ratios, making them much more difficult for models to detect and recognize. Classical object detection models are usually divided into two categories: anchor-based [10,11,12] and anchor-free [13,14,15,16]. Generally, the anchor-based models preset a set of anchors for regression to the object boundary by calculating the distribution of object scales in the training set. The performance of this type of model is very sensitive to hyperparameters such as the scale, size, and number of anchors, and it usually obtains better accuracy after debugging but also faces high computational complexity and delay. In addition, anchor-based models often struggle to return accurate boundaries for objects with extreme aspect ratios. The anchor-free models have gained wide attention due to the simplicity of their architecture. Methods such as corner points and center points do not rely on preset anchors and can be flexibly applied to various scenarios. Their detection accuracy is gradually approaching that of anchor-based models. Considering the advantages and disadvantages of the above models and the features of the signal object in the time–frequency domain, different detection schemes are proposed from the perspectives of the frequency centerline [17], frequency center point [18], and time start–stop point [19], which improves the adaptability of the model to the signal object to a certain extent. Therefore, it is essential to design a model that is adapted to the features of the signal object.

Based on the aforementioned research background, the core motivation of this study lies in targeted addressing of the key technical gaps existing in the current WSDR field, which are specifically reflected in the following three aspects: First, although existing methods have adapted to the extreme-aspect-ratio characteristics of signal targets to a certain extent, they fail to fully explore the inherent horizontally banded structures and long-range dependencies of time–frequency-domain signals, leading to feature extraction being susceptible to local noise interference. Second, most data augmentation strategies have directly migrated from the general object detection field, which may lead to performance degradation due to differences in domain characteristics. Third, the post-processing stage lacks the effective utilization of time–frequency distribution priors for signals. It is important to clarify that the time–frequency distribution priors referred to herein are not the signal-specific parameter priors relied on by traditional methods (e.g., modulation scheme, carrier frequency, cyclic frequency, symbol rate, etc.), but rather the universally existing spatial distribution patterns of signals in the time–frequency domain (such as energy presenting a horizontal band-like distribution, consistent frequency and bandwidth across multiple burst states of the same signal, etc.). Such priors possess universality and can guide the model to refine detection results. Therefore, designing a WSDR method that is deeply adapted to the characteristics of time–frequency-domain signals, integrates domain priors, and optimizes the feature extraction and post-processing processes became the core starting point of this study. In addition, a key limitation of more research in this area is the lack of openly available datasets. Fortunately, to encourage broader research on wideband operations, Boegner et al. [20] open-sourced the TorchSig algorithm package for simulating complex signals under wideband conditions. They have also introduced a large-scale wideband signal benchmark dataset, WidebandSig53 (WBSig53), for research related to the WSDR task. To the best of our knowledge, there is currently limited research on the WBSig53 dataset. Inspired by CenterNet and considering the characteristics of signal objects that exhibit horizontally oriented band-like structures in the time–frequency domain, we propose a WSDR method based on time–frequency distribution priors and structure-aware adaptivity. Our main contributions are as follows:

- We analyze the differences in the effectiveness of various data enhancement methods used for WSDR and propose a data enhancement scheme adapted to detect and recognize signals from the time–frequency domain based on the results of an ablation experiment.
- In view of the shape and distribution of signal objects in time–frequency domain, we introduce the Strip Pooling Module (SPM) and Coordinate Attention (CA) mechanism during the feature extraction and fusion stages, which help the model to aggregate the long-distance dependencies in the horizontal and vertical directions, avoiding the noise interference outside the local window, and thus better focusing on the spatial distributions and shape features of the signal. In addition, we employ an adaptive elliptical Gaussian coding strategy to generate heatmaps, which enhances the adaptability of the effective guidance region for center-point localization to the target shape.
- In the wideband spectrum, signal objects with same center frequencies and bandwidths can be considered as multiple burst states of the same signal. Based on this cognitive prior, we design a post-processing algorithm called the Time–Frequency Clustering Optimizer (TFCO) to assist model decision-making, aiming to improve the model’s recognition accuracy.
- Our approach is evaluated based on the WBSig53 dataset. The experimental results indicate that the proposed method achieves improved detection and recognition performance while maintaining low complexity compared to other existing methods.

The remainder of this paper is organized as follows: In Section 2, we introduce some related works on deep learning models for WSDR, along with current object detection methods. Section 3 introduces the proposed TFDP-SANet network architecture and TFCO post-processing algorithm. Section 4 discusses the setup and the experimental results. Finally, Section 5 concludes this paper.

## 2. Related Work

### 2.1. WSDR Based on Deep Learning

We have reviewed relevant work on WSDR research based on deep learning. Due to the differences in research subjects, there are variations in technical implementations. To clearly present the characteristics and distinctions of existing studies, we have organized related works into Table 1. Differently from the existing studies in the table, this paper aims to take the time–frequency-domain signal characteristics and domain prior knowledge as the entry point, and is committed to better adapting and applying the general object detection model to the downstream tasks of WSDR. For one-dimensional sequences such as sampled signals or power spectra, many works [21,22,23] have focused more on detecting signals and their bandwidths within the wideband spectrum, generally requiring the combination of filtering and intelligent modulation recognition models for further signal classification. In contrast, the two-dimensional representation in the time–frequency domain can reflect more information, such as time periods and time–frequency relationships. A commonly used time–frequency analysis method is the Short-Time Fourier Transform (STFT). Compared to other time–frequency analysis methods, such as nonlinear and bilinear time–frequency methods, STFT is a single-time–frequency-resolution method, which is computationally simple and does not produce cross-term interference. Some early works [24,25] attempted to detect signals and achieve time–frequency localization in the wideband spectrum using classical networks such as YOLO and Faster R-CNN, but do not consider modulation classification. Zha et al. [26] explored the problem of deep learning-based multi-signal detection and modulation classification for the first time. For M-ary Phase Shift Keying- and M-ary Quadrature Amplitude Modulation-modulated signals in the same regime that are difficult to distinguish in time–frequency images, they use filtering and down-conversion techniques to obtain the baseband signals, and then train the recognition network for modulation classification. The experimental results show that the method is more accurate in estimating the carrier frequency, but there is a large error in estimating the start–end time of the signal. This may be due to the fact that signal objects in time–frequency images have more extreme aspect ratios, unlike natural images. In this regard, Li et al. [17] argue that signal objects are usually beyond the receptive field of the feature map, which may lead to incomplete prediction boundaries for object detection algorithms based on the object center. Therefore, they suggest using the centerline to locate the signal and to classify and regress the signal bandwidth based on the features of the centerline. This approach discards the candidate anchor and predicts the signal bandwidth by establishing a centerline-connected domain through multiple object pixel points, which is more relevant to the task orientation. Furthermore, Li et al. [18] propose a new deep learning framework for high-frequency signal detection in the wideband spectrum. The framework integrates all the features along the time axis and predicts a signal object bounding box for each frequency point, achieving a more concise and efficient signal detection and recognition network. However, there may be a risk of missed detection for signal objects with overlapping center frequencies but different bandwidths and types. Cheng et al. [19] propose a start–stop point CenterNet, which achieves excellent performance in terms of accuracy and computational complexity. Compared to the centerline method, the start–stop method requires a smaller number of parameters to be regressed, making it relatively less difficult to implement. However, this method may encounter difficulties in distinguishing between signals with similar low or high frequencies but different bandwidths and modulation types, and it also fails to capture the multiple burst durations of discontinuous burst signals. Li et al. [27] proposed an Anchor-Free SNR-Aware signal detector, which adapts to the characteristics of wideband signals through an anchor-free architecture and enhances detection robustness in complex noise environments by integrating SNR awareness. In addition, to address the significant degradation of signal detection performance in unknown high-frequency channels, Lin et al. [28] propose a novel multi-resolution signal detection and classification network incorporating a domain-adaptive approach. The network is used for real-time multi-signal detection and classification in known and unknown channels, offering a new solution for deployment in practical environments.

### 2.2. Object Detection Model

In recent years, convolutional and transformer architectures have dominated the field of computer vision models. The transformer architecture, with its powerful global modeling capabilities, has shown unique advantages in object detection tasks. Transformer-based object detection models have primarily evolved along two lines: Detection Transformer [29] and Vision Transformer [30]. However, due to the lack of inductive biases, such as translational invariance and locality, these models typically require large amounts of data and longer training times to achieve optimal performance. In contrast, convolutional neural networks, thanks to their inherent inductive biases, can converge quickly on relatively small datasets and demonstrate good generalization capabilities. Especially in specific scenarios and practical deployment applications, convolutional architectures show obvious advantages. In recent work, anchor-free models have been favored for their simple yet effective designs, such as CornerNet [31], FCOS [13], CenterNet [16], and others. These models simplify their structure by defining the positions of objects (e.g., corner points, center points) rather than relying on predefined anchor boxes, achieving a good balance between accuracy and efficiency. While transformer architectures have introduced new possibilities for visual tasks, convolutional architectures continue to hold an important position in many practical applications due to their efficiency, stability, and ease of optimization.

## 3. Proposed Method

The WSDR task aims to detect and recognize multiple heterogeneous signals within a wideband spectrum by estimating four key parameters for each signal: (1) modulation type, (2) bandwidth, (3) start and end time, and (4) center frequency. In this section, we describe how the model can be adapted to the WSDR task through data enhancement strategies, network structures, coding strategies, and post-processing algorithms.

### 3.1. Data Augmentation Strategy

**Figure 1:**
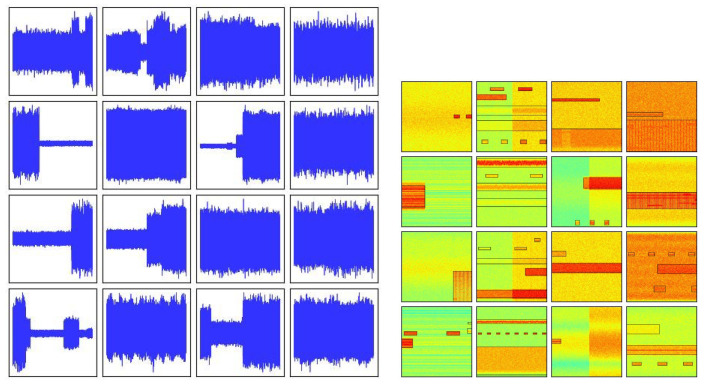
Time-domain waveform and STFT image of WBSig53 dataset.

Appropriate data augmentation strategies are crucial for expanding the diversity of task datasets, as they enable the model to capture general cognitive patterns from the data, thereby significantly improving the robustness and generalization ability of the model. Data augmentation strategies must be tightly coupled with the characteristics of the dataset and designed based on real-world properties; otherwise, they may backfire and impair the performance of the model. Common types of data augmentation include geometric transformations, color space transformations, and mixed transformations. In general object detection tasks, CenterNet applies a series of data augmentation techniques including random flip, random scaling, cropping, and color jittering to the COCO2017 dataset. This study is mainly carried out on the WBSig53 dataset. The time-domain waveforms and STFT example diagrams of the signals in the dataset are shown in Figure 1.

Through in-depth analysis of the characteristics of the WBSig53 dataset and combining the results of the ablation experiments, we find that the color space transformation did not enhance the model’s recognition ability, but rather weakened the discriminative power of the model. This is mainly because the images in the WBSig53 dataset are derived from signal time–frequency transformation, where the color information essentially encodes the amplitude and frequency distribution characteristics of the original signal—rather than the natural visual color features that color space transformation is designed to optimize. Blindly applying traditional color space transformation disrupts the inherent correlation between the signal-derived color channels. In addition, it is still a challenging task to detect and recognize small objects in time–frequency images. Based on these findings, we propose a set of data augmentation strategies that are particularly suitable for the WBSig53 dataset—including horizontal flipping, random cropping, random scaling, and mosaic augmentation—which are effective in improving the model’s adaptability and recognition of the WBSig53 dataset.

### 3.2. Network Structure

**Figure 2:**
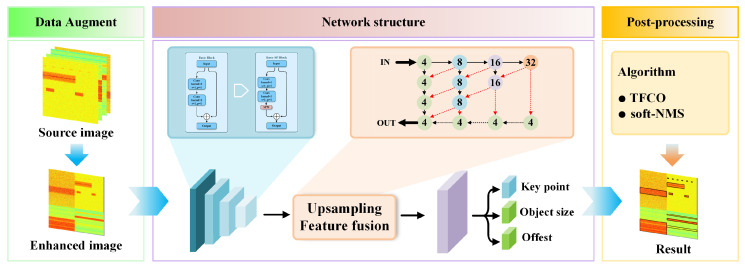
Schematic of TFDP-SANet method.

As shown in Figure 2, the network structure includes a feature extraction layer, upsampling fusion layer, and regression branch. We can use it to predict the keypoints $\hat{Y}$, offset $\hat{O}$, and size $\hat{S}$. This paper optimizes the network architecture by refining two critical components—feature extraction and upsampling fusion layers—to enhance its capability in focusing on signal object features within time–frequency images.

**Figure 3:**
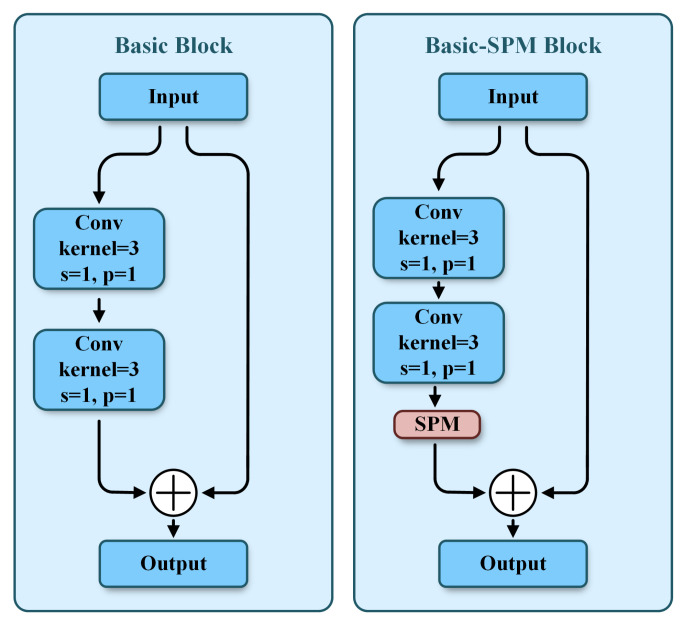
Schematic of the Basic block and Basic–SPM block.

First, we select Deep Layer Aggregation 34 (DLA34) as the feature extraction network for 4× downsampling. Classic deep networks such as ResNet and EfficientNet mainly focus on optimizing the deep propagation process of feature extraction, and usually require additional structures like a Feature Pyramid Network and Path Aggregation Network to achieve feature fusion across different levels. In contrast, DLA34 inherently incorporates an iterative aggregation mechanism for multi-level feature information. This characteristic enables it to combine feature extraction and fusion within a unified framework, achieving higher accuracy with fewer parameters. By integrating semantic fusion and spatial fusion, it further enhances the ability to recognize objects and their locations. The foundational component of DLA34 is the Basic block. Considering the limited receptive field of traditional convolutions and their weak ability to capture long-range dependencies, common practices involve using dilated convolutions, global pooling, and pyramid pooling to expand the receptive field and capture global information. However, these methods are not sufficiently effective for slender signal objects. The SPM [32] has been better applied in semantic segmentation. It utilizes horizontal and vertical strip pooling operations to collect long-range context from different spatial dimensions and achieves input feature refinement by encoding location information. Distinct from conventional square pooling, strip pooling averages the feature values across each row or column, thereby circumventing the inclusion of noise from regions that are not associated with the slender signal objects. Thus, we propose to build the Basic–SPM block by integrating the SPM into the Basic block (see Figure 3). By reweighting the input, the SPM enhances the Basic block’s local perception and global understanding of the input feature in both horizontal and vertical directions.

**Figure 4:**
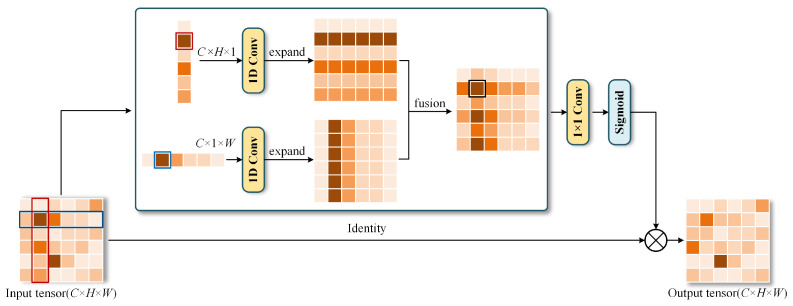
Schematic illustration of the SPM.

The SPM is depicted in Figure 4. Specifically, given the tensor m$\in R^{C\times H\times W}$, *H* and *W* are the spatial height and width, and *C* denotes the number of channels. Strip pooling averages all the feature values in each row or column along the horizontal and vertical directions to capture long-range spatial dependencies.

For horizontal strip pooling, it averages all feature values for each row, producing a horizontally pooled tensor $n^{h}\in R^{C\times H\times 1}$. The mathematical formulation is

(1) $$n_{c,i}^{h}=\frac{1}{W}\sum_{0\leq j<W}m_{c,i,j}.$$

where $n_{c,i}^{h}$ denotes the horizontally pooled value of the c-th channel at the i-th row, and $m_{c,i,j}$ is the feature value of the input tensor m at position (c,i,j). Similarly, vertical strip pooling averages feature values for each column, generating a vertically pooled tensor $n^{w}\in R^{C\times 1\times W}$. Its definition is

(2) $$n_{c,j}^{w}=\frac{1}{H}\sum_{0\leq i<H}m_{c,i,j}.$$

where $n_{c,j}^{w}$ represents the vertically pooled value of the c-th channel at the j-th column. To exploit the pooled global information, we apply 1D convolutions to $n^{h}$ and $n^{w}$, respectively, which modulate the feature responses of local and neighboring regions. We then expand their shape to match the input. To obtain more useful global priors, we combine ${\hat{n}}^{h}\in R^{C\times H\times W}$ and ${\hat{n}}^{w}\in R^{C\times H\times W}$ together, yielding z$\in R^{C\times H\times W}$.

(3) $$z=\mathrm{Scale}(m,\sigma \left(f\left({\hat{n}}^{h}+{\hat{n}}^{w}\right)\right)).$$

where Scale(·, ·) is the element-wise multiplication, σ is a sigmoid function, and *f* is a 1 × 1 convolutional transformation.

Furthermore, it is a challenge to improve the network’s attention on object location information in multi-scale features. In recent years, attention mechanisms based on plug-and-play characteristics have been widely used in computer vision and achieved significant results. Particularly, the CA [33] mechanism, which simultaneously enhances the representation of channel and spatial information, is more suitable for the vision task of capturing object structure than attention mechanisms such as squeeze-and-excitation and the convolutional block attention module.

**Figure 5:**
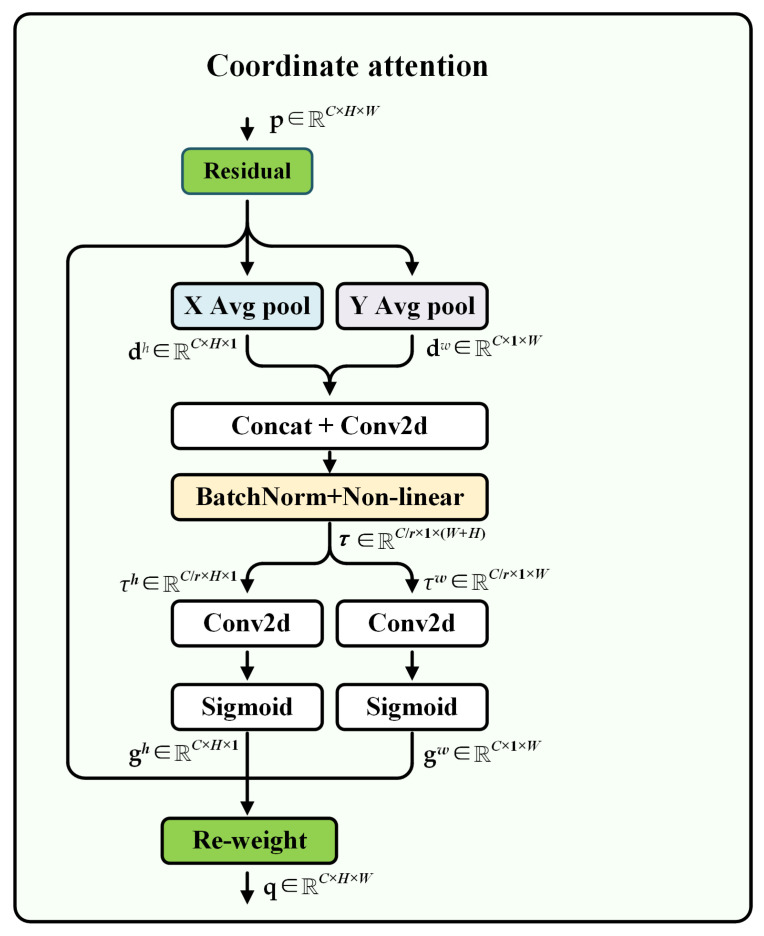
Schematic illustration of the CA module.

Therefore, during the feature fusion and upsampling stages, we combine the CA mechanism with deformable convolution to enhance the network’s extraction of spatial features at different scales. By focusing on critical spatial information across scales, the CA improves the model’s multi-scale perception capability, leading to more precise object localization and recognition. This module is highly lightweight and achieves significant performance improvements with minimal computational overhead. Specifically, CA performs average pooling operations on the input in the horizontal and vertical directions, respectively, aggregating them into two direction-aware feature maps. Then, by concatenating these two feature maps and conducting convolutional operating, attention weights corresponding to the respective directions are generated. The attention weights are fused with the original input features through weighted combination, thereby enhancing the feature representation of the regions of interest. The computational process of the CA module is shown in Figure 5. Given the tensor $p\in R^{C\times H\times W}$, like the SPM, we first use horizontal and vertical strip pooling to get $d^{h}\in R^{C\times H\times 1}$ and $d^{w}\in R^{C\times 1\times W}$. They enable CA to capture long-range dependencies and help networks locate objects more accurately.

Then, we concatenate them along the spatial dimension and send them to a shared 1 × 1 convolutional transformation $F_{1}$, a BatchNorm layer, and a nonlinear activation function δ, yielding

(4) $$\tau =\delta (F_{1}\left(\left[d^{h},d^{w}\right]\right)).$$

where $\tau \in R^{\frac{C}{r}\times 1\times \left(H+W\right)}$ is the intermediate feature map, and *r* is a reduction ratio used to reduce the complexity of the model. Next, τ is divided into two tensors $\tau^{h}\in R^{\frac{C}{r}\times H\times 1}$ and $\tau^{w}\in R^{\frac{C}{r}\times 1\times W}$ along the spatial dimension. The numbers of channels for $\tau^{h}$ and $\tau^{w}$ are transformed to be the same as the input p using two 1 × 1 convolutional transformations $F_{h}$ and $F_{w}$, yielding

(5) $$g^{h}=\sigma \left(F_{h}\left(\tau^{h}\right)\right).$$

(6) $$g^{w}=\sigma \left(F_{w}\left(\tau^{w}\right)\right).$$

where σ is a sigmoid function, and $g^{h}\in R^{C\times H\times 1}$ and $g^{w}\in R^{C\times 1\times W}$ are used as attention weights, respectively. At last, the output $q\in R^{C\times H\times W}$ of the CA module can be defined as

(7) $$q_{c,i,j}=p_{c,i,j}\times g_{c,i}^{h}\times g_{c,j}^{w}.$$

### 3.3. Adaptive Elliptic Gaussian Encoding Strategy

When the aspect ratio of signal targets is large, the central guidance region of circular Gaussian encoding exhibits a mismatch with the actual shape of the targets. To address this issue, we propose an adaptive elliptical Gaussian encoding strategy. This strategy enables the guidance range of the loss function to better conform to the shape characteristics of the targets, thereby improving the accuracy and robustness of the model in locating center points in scenarios with diverse aspect ratios. Let $I\in R^{3\times H\times W}$ be an input image. The objective is to generate a keypoint heatmap $\hat{Y}\in {\left[0,1\right]}^{C\times \frac{H}{k}\times \frac{W}{k}}$, where *k* is the output stride and *C* is the number of keypoint classes. In the heatmap, a value of ${\hat{Y}}_{x,y,c}=1$ indicates the detection of a keypoint at the corresponding position (x,y) for the *c*-th channel, while ${\hat{Y}}_{x,y,c}=$ 0 implies that the location belongs to the background.

**Figure 6:**
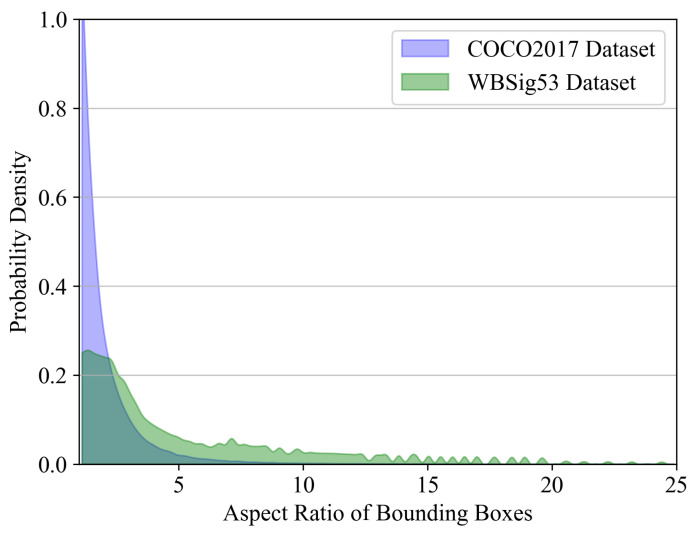
Bounding box aspect ratio distributions in COCO2017 and WBSig53 datasets.

However, such demanding dense prediction problems pose a great challenge for network learning. To reduce the difficulty of estimating the object center in the keypoint regression branch, CenterNet employs an encoding strategy in the label heatmap that constructs Gaussian distributions on the objects’ center. By optimizing the keypoint estimation loss function under this distribution, the convergence speed of the network is enhanced. Specifically, Zhou et al. [16] consider three scenarios regarding the relationship between the ground truth and the bounding box: inclusion, being included, and overlapping. In these scenarios, they determine the minimum radius *r* of the Gaussian circle that meets the IoU threshold. The label heatmap is downsampled by k= 4× in comparison to the input size. In the label heatmap, for each keypoint $u\in R^{2}$ belonging to class *C*, its coordinates are denoted as $\tilde{u}=\left\lfloor \frac{u}{k}\right\rfloor$ in the label heatmap. Subsequently, the Gaussian kernel function $Y_{x,y,c}=\exp\left(-\frac{{\left(x-{\tilde{u}}_{x}\right)}^{2}+{\left(y-{\tilde{u}}_{y}\right)}^{2}}{2\sigma_{p}^{2}}\right)$ is employed to distribute all the keypoints across the heatmap $Y\in {\left[0,1\right]}^{C\times \frac{H}{k}\times \frac{W}{k}}$, where $\sigma_{p}$ is the standard deviation related to *r*. However, the basic circular Gaussian kernel may be insufficiently guided in terms of the loss function for object center estimation at large aspect ratios. As shown in Figure 6, we analyze the aspect ratio distribution in both the COCO2017 and WBSig53 datasets, revealing a more pronounced imbalance in the object aspect ratio within the WBSig53 dataset.

**Figure 7:**
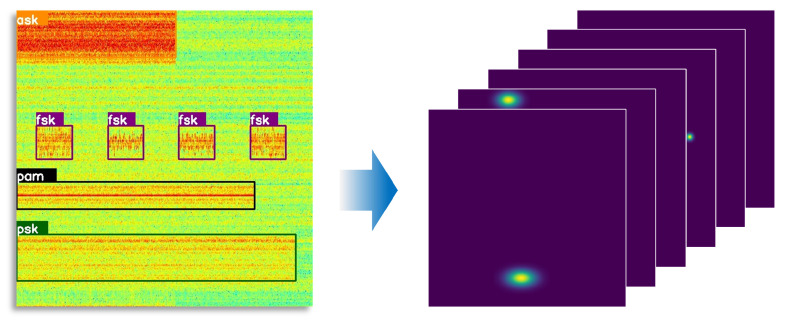
Time–frequency image and corresponding adaptive elliptic Gaussian kernel heatmap.

Thus, we propose the adaptive elliptic Gaussian kernel combined with the aspect ratio of the object (see Figure 7), which can be defined as

(8) $$Y_{x,y,c}=\exp\left(-\frac{{\left(x-{\tilde{u}}_{x}\right)}^{2}}{2\sigma_{x}^{2}}-\frac{{\left(y-{\tilde{u}}_{y}\right)}^{2}}{2\sigma_{y}^{2}}\right).$$

where $\sigma_{x}$ and $\sigma_{y}$ are standard deviations related to *r* and the aspect ratio. The range of the elliptical Gaussian distribution corresponds to that of a concentric rectangle, with its sides $r_{h}$ and $r_{w}$ defined as follows:

(9) $$\left(r_{h},r_{w}\right)=\left\{\begin{array}{cc} \left(r,r\times \left(1+log_{10}\left(\frac{w}{h}\right)\right)\right), & \mathrm{if}\;h\leq w\\ \left(r\times \left(1+log_{10}\left(\frac{h}{w}\right)\right),r\right), & \mathrm{if}\;h>w \end{array}\right.$$

where *h* and *w* are the size of the ground truth.

### 3.4. Loss Function

The loss function of TFDP-SANet consists of three weighted components: keypoint prediction loss $L_{hm}$, size loss $L_{size}$, and center offset loss $L_{off}$. Specifically, $L_{hm}$ is defined as follows:

(10) $$L_{hm}=\frac{-1}{N}\sum_{xyc}\left\{\begin{array}{cc} {\left(1-{\hat{Y}}_{x,y,c}\right)}^{\alpha }\log\left({\hat{Y}}_{x,y,c}\right), & \mathrm{if}\;Y_{xyc}=1\\ {\left(1-Y_{x,y,c}\right)}^{\beta }{\left({\hat{Y}}_{x,y,c}\right)}^{\alpha } & \\ \log(1-{\hat{Y}}_{x,y,c}), & \mathrm{otherwise} \end{array}\right.$$

where *N* denotes the number of keypoints, and α=2 and β=4 are hyperparameters [11]. $L_{hm}$ adds a decay term to the Focus loss, weakening the loss of negative samples within the adaptive elliptical Gaussian kernel distribution. This prevents the model from overfocusing on negative samples and makes the model training more stable.

$L_{size}$ is calculated for all keypoints in image I. We use a single size prediction $\hat{S}\in R^{2\times \frac{H}{k}\times \frac{W}{k}}$ for all object classes. We define the bounding box size $s_{t}=(w_{t},h_{t}$ and center point $v_{t}=\left(x_{t},y_{t}\right)$ of object *t* in image I. Then, they need to be converted to ${\tilde{s}}_{t}=\left\lfloor \frac{s_{t}}{k}\right\rfloor$ and ${\tilde{v}}_{t}=\left\lfloor \frac{v_{t}}{k}\right\rfloor$. We use the L1 function to calculate the loss:

(11) $$L_{size}=\frac{1}{N}\sum_{t=1}^{N}\left|{\hat{S}}_{t}-{\tilde{s}}_{t}\right|.$$

Due to the downsampling process of the feature map causing discretization errors at the center points compared to the original input, we design a local offset $\hat{O}\in R^{2\times \frac{H}{k}\times \frac{W}{k}}$ to correct this deviation which is shared across all classes. Here, we still use L1 loss to compute the center offset loss:

(12) $$L_{off}=\frac{1}{N}\sum_{t=1}^{N}\left|{\hat{O}}_{t}-\left(\frac{v_{t}}{k}-{\tilde{v}}_{t}\right)\right|.$$

We do not employ normalization operations in the computation of $L_{size}$ and $L_{off}$. Instead, we directly weight and combine the loss from the three parts to obtain the total loss $L_{det}$ as follows:

(13) $$L_{det}=L_{hm}+\lambda_{size}L_{size}+\lambda_{off}L_{off}.$$

where $\lambda_{size}$ = 0.1 and $\lambda_{off}$ = 1 are weighting factors.

### 3.5. Post-Processing Algorithm

In wideband spectrum analysis, we note that signal objects with same center frequencies and bandwidths within a certain time window are usually multiple burst states of the same signal. However, the model tends to misclassify these burst states across different periods as distinct classes. In addition, the keypoint branch used a sigmoid function to output the heatmap, which does not significantly suppress the confidence scores of non-target categories for a keypoint. During inference, multiple-category prediction boxes corresponding to the same keypoint may be retained due to confidence levels exceeding the threshold. Since the Non-Maximum Suppression (NMS) algorithm is applied separately for each category, the situation cannot be avoided.

**Figure 8:**
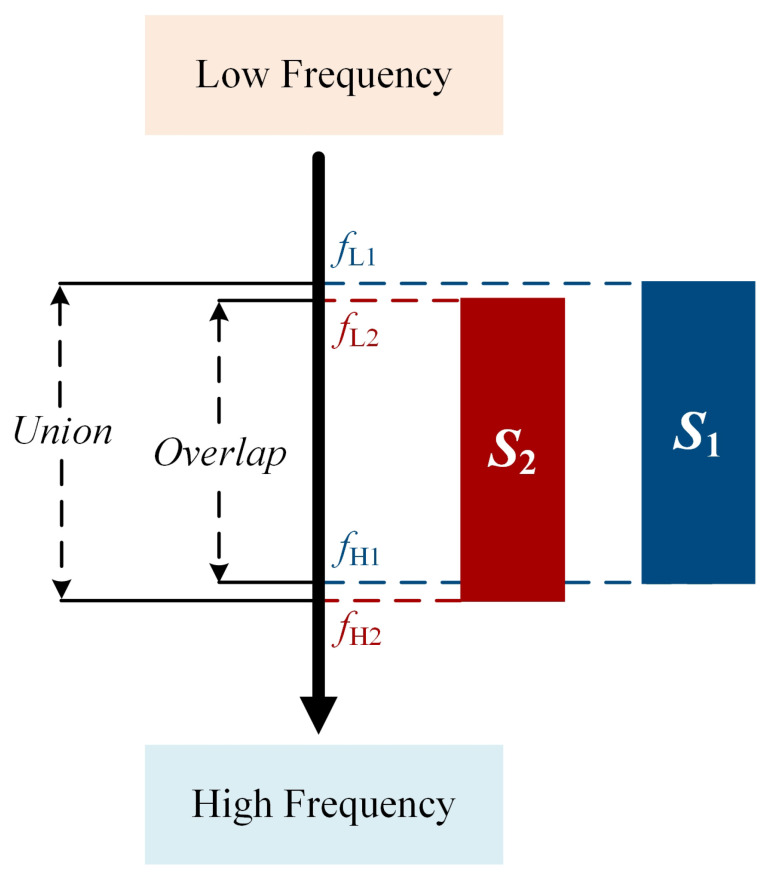
Schematic of the Interval-IOU.

Therefore, we propose a post-processing algorithm called the TFCO to alleviate the above problem. Typically, we sort the prediction boxes based on confidence and use the top *K* prediction boxes as the output of the model. Let $\left(x_{1}^{\left(i\right)},y_{1}^{\left(i\right)},x_{2}^{\left(i\right)},y_{2}^{\left(i\right)}\right)$ be the prediction box of object i∈K, where $y_{1}^{\left(i\right)}$ denotes low frequency $f_{L}^{\left(i\right)}$ and $y_{2}^{\left(i\right)}$ denotes high frequency $f_{H}^{\left(i\right)}$. In order to measure the similarity of *K* prediction boxes in center frequency and bandwidth, Interval-IoU is designed for evaluation. Inspired by the Intersection over Union (IoU) design in object detection, we present Interval-IoU, used to assess the proximity of signal frequency ranges. Interval-IoU is shown in Figure 8. Taking signals $S_{1}$ and $S_{2}$ as examples, with frequency ranges $\left(f_{L1},f_{H1}\right)$ and $\left(f_{L2},f_{H2}\right)$, the Interval-IoU formula is defined as follows:

(14) $$\begin{array}{cc} \; & \mathrm{Interval}\text{-}\mathrm{IoU}=\frac{\left(f_{L1},f_{H1}\right)\cap \left(f_{L2},f_{H2}\right)}{\left(f_{L1},f_{H1}\right)\cup \left(f_{L2},f_{H2}\right)} \end{array}$$

(15) $$\begin{array}{cc} \; & =\frac{\min\left(f_{H1},f_{H2}\right)-\max\left(f_{L1},f_{L2}\right)}{\max\left(f_{H1},f_{H2}\right)-\min\left(f_{L1},f_{L2}\right)} \end{array}$$

We compute the Interval-IoU between the *K* prediction boxes. Because narrow-bandwidth signals are more sensitive to changes in Interval-IOU than large-bandwidth signals, we designed a dynamic Interval-IOU threshold function δ(γ) to deal with this issue.

(16) $$\begin{array}{cc} \; & \delta \left(\gamma \right)=\gamma +\left(1-\gamma \right)\times \left(1+log_{2}\frac{b}{d}\right) \end{array}$$

where *b* represents the overlap of the frequency range, *d* denotes the bandwidth of the time–frequency image, and γ is a hyperparameter set to 0.8. We build a graph with prediction boxes as nodes. An edge is added between two nodes if their Interval-IOU on the y-axis exceeds a dynamic threshold. We use Breadth-First Search to find connected components in the graph. Each connected component represents a group of overlapping bounding boxes. For each group, we assign the category of the rectangle with the maximum confidence score to all members of the group, provided that the confidence exceeds a predefined threshold ρ= 0.8. In addition, for the case where the network outputs multiple prediction boxes of different categories at a single keypoint, these prediction boxes will be grouped into the same category after post-processing, because they are completely overlapping in position. The soft-NMS [34] algorithm can be used to retain only the prediction box with the maximum confidence. Details on the post-processing approach are presented in Algorithm 1.
**Algorithm 1** Time–Frequency Clustering Optimizer (TFCO)**Require:**   1:The set of prediction boxes output by the network $U=\{P_{i}|\left(x_{1}^{\left(i\right)},y_{1}^{\left(i\right)},x_{2}^{\left(i\right)},y_{2}^{\left(i\right)},confidence^{\left(i\right)},C^{\left(i\right)}\right),i\in K\}$;  2:The maximum confidence threshold, ρ;  3:The hyper-parameters of dynamic Interval-IOU threshold function, γ;**Ensure:**  4:**for** i=0 to K−1 **do**  5:    **for** j=i+1 to K−1 **do**  6:        IoU ← Interval-IoU($y_{1}^{\left(i\right)}$, $y_{2}^{\left(i\right)}$, $y_{1}^{\left(j\right)}$, $y_{2}^{\left(j\right)}$)  7:        Threshold ←δ($y_{1}^{\left(i\right)}$, $y_{2}^{\left(i\right)}$, $y_{1}^{\left(j\right)}$, $y_{2}^{\left(j\right)}$, γ)  8:        **if** IoU > Threshold **then**  9:            Add *j* to graph[*i*]10:            Add *i* to graph[*j*]11:        **end if**12:        **end for**13:**end for**14:Initialize an empty dictionary graph15:Initialize an empty set visited16:Initialize an empty list components17:**for** node=0 to k−1 **do**18:    **if** node∉ visited **then**19:        Initialize an empty list component20:        Initialize a deque queue with node21:        **while** queue is not empty **do**22:           current←popleftfromqueue23:           **if** current∉visited **then**24:               Add current to visited25:               Add current to component26:               **for all** neighbor∈graph[current] **do**27:                   **if** neighbor∉visited **then**28:                       Add neighbor to queue29:                   **end if**30:                **end for**31:            **end if**32:        **end while**33:        Add component to components34:     **end if**35:**end for**36:**for** component∈components **do**37:     Find the member with the highest confidence in the component:38:     **for** k∈component **do**39:        **if** $confidence^{\left(k\right)}>max\_confidence$ **then**40:           $max\_confidence\leftarrow confidence^{\left(k\right)}$41:           $best\_category\leftarrow C^{\left(k\right)}$42:        **end if**43:     **end for**44:     **if** max_confidence>ρ **then**45:        **for** k∈component **do**46:            Update the category of $P_{k}$ to best_category47:        **end for**48:     **end if**49:**end for**

## 4. Experiments

This section presents a series of experiments designed to assess the performance of our proposed model using the WBSig53 dataset. We first describe the dataset, training scheme, and evaluation metrics in detail. Subsequently, we conduct thorough ablation studies to validate the efficacy of our method and benchmark its performance against other advanced object detection methods. All results are reported on an NVIDIA A100 GPU with Pytorch2.0.1.

### 4.1. Implementation Details

In this section, we provide a comprehensive overview of the implementation details. Specifically, we describe the WBSig53 dataset used for training and evaluation, define the evaluation metrics adopted to quantify performance, and outline the training scheme. The configuration parameters related to the dataset and training procedure are consolidated into Table 2.

#### 4.1.1. WBSig53 Dataset

This dataset was released by the TorchSig team in 2023 and consists of 550,000 signal examples encompassing around 2 million signal instances divided into four distinct subsets: Clean Training (250,000 examples), Clean Validation (25,000 examples), impaired training (250,000 examples), and impaired validation (25,000 examples). The WBSig53 covers six modulation families: ask, pam, psk, qam, fsk, and ofdm. To more realistically simulate real-world scenarios, the generation of the impaired dataset applies 12 diverse methods to emulate real radio-frequency impairments on complex-valued I/Q samples. This paper employs the benchmarking experimental setup published by TorchSig, demonstrating the effectiveness of the proposed methods by training and validating them on the impaired dataset in modulation family classes. Each signal sample in the dataset is a 262,144-length I/Q data point. According to the STFT parameters predefined by TorchSig, we maintain an FFT window length of 512, with a Blackman window function and no window overlap. The resulting 512 × 512 complex-valued STFT matrix is saved as an image for network input.

#### 4.1.2. Evaluation Metrics

In order to further illustrate the effectiveness of the method described in this paper, we conduct a comparison with other models based on Mean Average Precision (mAP), Mean Average Recall (mAR), model parameters (Params), Floating-Point Operations (FLOPs), and Frames Per Second (FPS). Specifically, precision (P) and recall (R) are first defined: precision measures the proportion of true positive samples among all detected positive results, calculated as $P=\frac{TP}{TP+FP}$; recall measures the proportion of true positive samples correctly detected among all actual positive samples, formulated as $R=\frac{TP}{TP+FN}$. Here, true positive (TP) denotes the number of prediction boxes with IoU ≥ the set threshold and correct classification; False Positive (FP) represents the number of falsely detected prediction boxes; and False Negative (FN) is the number of actual targets that fail to be correctly detected. Average Precision (AP) is defined as the integral of the P-R curve over the interval [0,1], computed as AP $=\int_{0}^{1}P\left(r\right)dr$, where P(r) is the interpolated precision function and r denotes recall. mAP is the average of APs across all target classes, with the formula mAP $=\frac{1}{C}\sum_{c=1}^{C}AP_{c}$, where C is the number of target classes and $AP_{c}$ is the AP value of the c-th class. Additionally, we provide results for AP_50_ and AP_75_ (corresponding to AP values at IoU thresholds of 0.5 and 0.75, respectively), as well as AP_*s*_, AP_*m*_, and AP_*l*_ (AP values for small objects with area $<32^{2}$ pixels, medium objects with $32^{2}\leq$ area $\leq 96^{2}$ pixels, and large objects with area $>96^{2}$ pixels, respectively) to demonstrate the model’s performance across different object scales and IoU thresholds. mAR further reflects the network’s detection capability for signal targets, which is of great significance in specific spectral monitoring scenarios. Its mathematical expression is mAR $=\frac{1}{C}\sum_{c=1}^{C}AR_{c}$, where $AR_{c}$ is the average recall of the c-th class across multiple IoU thresholds. Both Params and FPS serve as critical references for the practical deployment of the model. The computation of FPS adheres to the guidelines of MMDetection, encompassing model forward passes and post-processing steps. For accurate FPS measurements, all results are obtained from inference runs on a single NVIDIA A100 GPU.

#### 4.1.3. Training Scheme

The WBSig53 impaired training data serves as the experimental training set, comprising 250,000 images, while the impaired validation set functions as the experimental validation set, consisting of 25,000 images. The test set is identical to the validation set. We set the batch to 128 and epoch to 140. The initial learning rate is set to 1 × 10^−4^ and is decayed at the 90th and 120th epochs. To improve accuracy and convergence, we use pre-trained weights. During inference, we set the maximum number *K* of objects per image to 100 and a confidence threshold of 0.3, and combined post-processing algorithms with soft-NMS to obtain the final predicted results.

### 4.2. Ablation Experiment

We employ CenterNet as the baseline network and conduct ablation experiments from four perspectives—data augmentation, network structure, encoding strategy, and post-processing algorithms—to validate the effectiveness of the proposed method.

#### 4.2.1. Appropriate Data Augmentation Strategy Is Crucial

As shown in Table 3, effective color space transformations, such as adjustments to brightness, contrast, saturation, and lighting, which are commonly applied in natural image detection, are not suitable for time–frequency images. This is mainly due to the fact that time–frequency images are intrinsically unaffected by these factors in the real world. In contrast, techniques like flipping, scaling, cropping, and mosaic augmentation match the actual scene and can be effectively used to enrich the diversity of samples. Specifically, flipping improves mAP by 1.1%, while scaling and cropping improve the mAP by 1.9%. However, color space changes result in a reduction in mAP by 0.7%. Mosaic augmentation combined with flipping, scaling, and cropping to integrate four samples into one sample significantly improves the mAP by 2.6%. This data augmentation strategy significantly enhances the overall performance of the WSDR task.

#### 4.2.2. Focusing on Structural Features Is Beneficial

In the feature extraction stage, integrating the SPM into the Basic block achieves higher-quality spatial feature aggregation and effectively improves the network’s ability to focus on objects. As shown in Table 4, the Basic–SPM block achieves the most significant performance improvement, with a 0.9% increase in mAP. This enhancement results in a rise in model parameters and a reduction in FPS. In the upsampling and feature fusion stages, we propose the utilization of the CA mechanism combined with deformable convolution to strengthen the extraction of key features. This approach leads to a 0.3% improvement in mAP without significantly increasing the number of network parameters. Additionally, the elliptic Gaussian coding strategy enhances the mAP by 0.2% without imposing additional computational load on the model during inference.

#### 4.2.3. Incorporating Prior Knowledge Reasonably Is Feasible

The WSDR task has special characteristics compared to generic object detection. In general, we argue that multiple signals with the same center frequency and bandwidth within a short time window are burst states of the same signal. Based on this assumption, we design the TFCO algorithm to further adjust the class of bounding boxes. However, the TFCO algorithm relies on the model’s current level of understanding, so it needs to be utilized with great caution. The benefits of this method are more pronounced for high-performance models, while it may be counterproductive for models with average performance. We propose three approaches for implementing class adjustment. For bounding boxes grouped by Interval-IoU, we have calculated the cumulative sum of confidence scores, frequency of occurrence, and the maximum confidence score for each class. The experimental results in Table 5 show that the maximum confidence value is more effective in correcting the model’s class misjudgments compared to the other two statistics. To further minimize the propagation of errors, we only perform class adjustment on groups where the maximum confidence is greater than ρ=0.8. As shown in Table 5, combining the soft-NMS algorithm with the TFCO improves the mAP by 0.2%.

### 4.3. Comparative Experiment

To demonstrate the effectiveness of the TFDP-SANet method, we select several advanced object detection models as benchmarks for comprehensive comparative experiments. Specifically, the evaluation results of all models on the WBSig53 dataset are shown in Table 6. Throughout both the training and inference processes, we maintain a consistent input size of 512 × 512 time–frequency images for all networks. Moreover, we also present the baseline experiment results provided by the TorchSig team, which are marked with an asterisk (*) in Table 6. Experimental results demonstrate that the proposed method exhibits superior performance across the majority of metrics. In the comparative experiment, YOLOv3 demonstrates the fastest detection speed and also performs well in terms of mAP. Conditional-DETR achieves the best results in small-object detection and ranks second overall.

**Figure 9:**
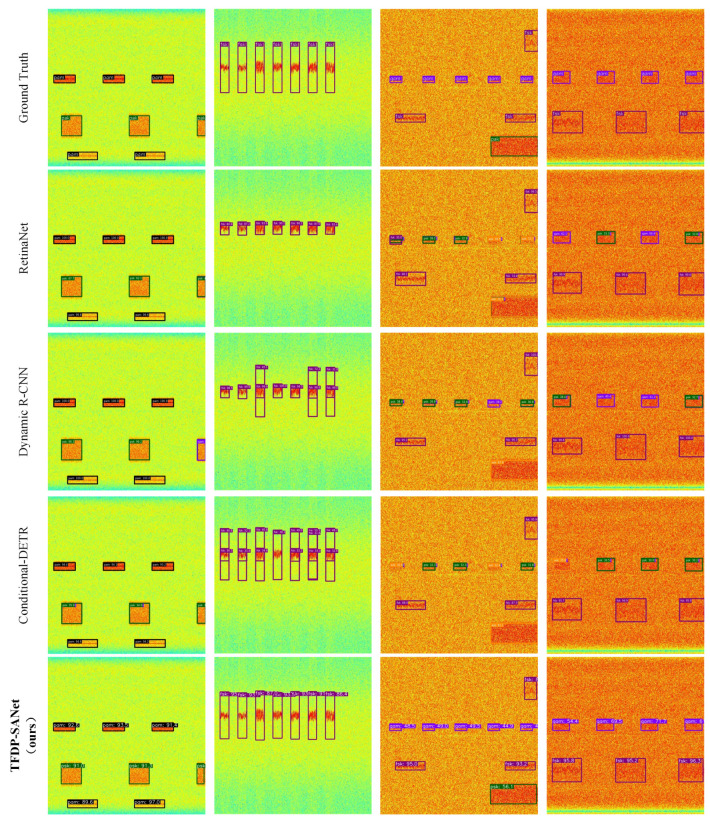
Visual examples of different object detection algorithms on the WBSig53 dataset: the orange, purple, blue, black, green, and magenta bounding boxes indicate that the detection results are ask, fsk, ofdm, pam, psk, and qam, respectively.

TFDP-SANet achieves the best performance across multiple AP metrics, while also maintaining a decent inference speed. In addition, we also enumerate the detection and classification results of all models on some samples from the WBSig53 validation set in Figure 9. Among them, we can observe two phenomena. Firstly, most models tend to misclassify the categories when dealing with multiple burst signals from the same emission source. TFDP-SANet using the TFCO algorithm can improve this situation. Secondly, TFDP-SANet exhibits superior capabilities compared to alternative models in terms of accurately regressing the boundaries of signal instances.

**Figure 10:**
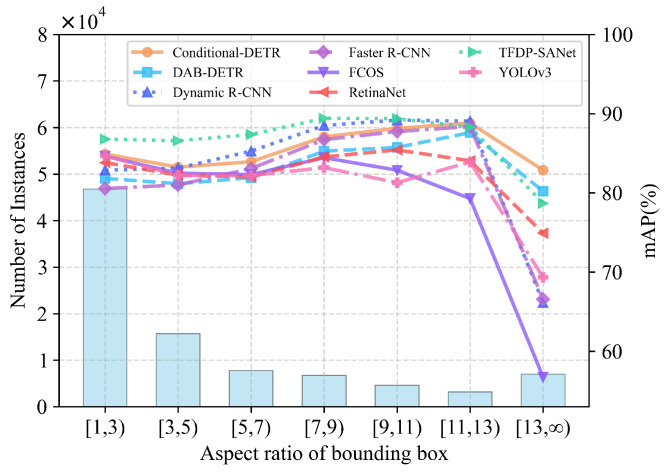
Comparison of mAP and instance distribution of different models under various bounding box aspect ratios.

To verify the unique advantages of TFDP-SANet in target structure perception, we statistically analyzed the mAP performance of various models for targets across different aspect ratio intervals, with specific results shown in Figure 10. As can be seen from the figure, the distribution of target aspect ratios exhibits significant differences, covering a full range of scenarios from compact forms (aspect ratio ≤3) to extremely elongated forms (aspect ratio ≥13). The comparison results indicate that TFDP-SANet demonstrates stable performance advantages across all aspect ratio intervals: in the case of medium-form targets with aspect ratios ranging from 1 to 11, its mAP almost surpasses that of all other models; in the case of extremely elongated targets with aspect ratios ≥13, its mAP value is only lower than that of transformer-based models. Overall, these results fully confirm the strong adaptability of TFDP-SANet to changes in target structures.

**Figure 11:**
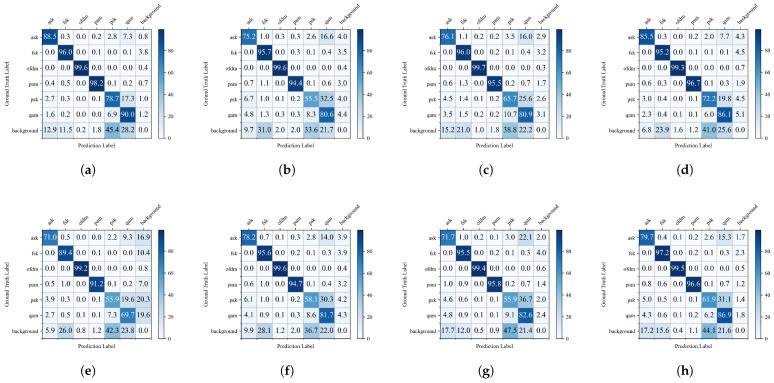
Confusion matrices of different models: (**a**) TFDP-SANet, (**b**) Faster R-CNN, (**c**) RetinaNet, (**d**) YOLOv3, (**e**) FCOS, (**f**) Dynamic R-CNN, (**g**) DAB-DETR, (**h**) Conditional-DETR.

To comprehensively analyze the classification performance of different object detection models, we present their confusion matrices in Figure 11, where a darker shade of blue in the grid indicates a higher recognition rate. It can be observed that all models exhibit varying degrees of misclassification among the three amplitude/phase modulation signal categories: ask, psk, and qam. However, TFDP-SANet demonstrates significantly superior performance in distinguishing features of these three signal types. Specifically, the recognition accuracies of TFDP-SANet for ask, psk, and qam signals reach 88.5%, 78.7%, and 90%, respectively. Compared with comparative models such as Faster R-CNN, DAB-DETR, and Conditional-DETR, the accuracy of each category is improved by an average of 5–10 percentage points. Overall, TFDP-SANet not only achieves excellent overall classification accuracy but also possesses lower inter-class confusion and a reduced background misclassification rate. In contrast, models such as Faster R-CNN, DAB-DETR, and Conditional-DETR show more pronounced misclassifications for amplitude and phase modulation signals like ask, psk, and qam. For multi-carrier modulation, frequency modulation, and pulse modulation signals, the average recognition rate of most models exceeds 95%. These results indicate that TFDP-SANet can effectively capture the fine-grained features of time–frequency images, achieving better classification accuracy and robustness in WSDR tasks.

### 4.4. Complexity Analysis

To comprehensively evaluate the complexity of TFDP-SANet, we conduct a comparative analysis with models such as Faster R-CNN, RetinaNet, YOLOv3, FCOS, Dynamic R-CNN, DAB-DETR, and Conditional-DETR from three core dimensions—Params, FPS, and FLOPs—under the same hardware environment (Intel 8358P CPU, NVIDIA A100 GPU) to ensure fairness of the comparison. It is important to clarify that the NVIDIA A100 GPU is only used for model training to guarantee training efficiency; the inference phase does not depend on such high-performance computing resources. As shown in Table 7, TFDP-SANet achieves an excellent balance across the three metrics: it has only 25.7 M parameters and a computational overhead of 26.9 GFLOPs. In terms of inference efficiency, its FPS is 47, second only to YOLOv3. The above experimental results fully demonstrate the superiority of TFDP-SANet in complexity and efficiency. Considering that devices such as IoT sensors and Systems-on-Chip generally suffer from limited hardware and computing capabilities, we have designed a set of adaptive optimization schemes for the inference phase. Specifically, INT8 model quantization is adopted to reduce memory footprint and latency without significant accuracy loss, and TensorRT operator acceleration is integrated to effectively improve the execution efficiency of edge hardware; meanwhile, lightweight edge inference frameworks such as ONNX Runtime are employed to accurately match the computing capabilities of the devices. These optimization measures work together to significantly enhance the deployability of the model in practical IoT scenarios.

## 5. Conclusions

In summary, we propose an effective TFDP-SANet for the WSDR task. First, we design a novel Basic–SPM block and integrate it with CA to enhance the ability to extract features in both horizontal and vertical directions, which is adaptive to the structural characteristics and spatial distribution of signal objects. Furthermore, the adaptive elliptical Gaussian encoding strategy optimizes the guidance effect of center-point localization by dynamically adapting to the signal shape, thereby reducing detection deviations caused by aspect ratio differences. The post-processing algorithm incorporating prior information can also improve the model’s ability to some extent. Under a similar network scale, the proposed method can achieve higher accuracy and maintain a faster inference speed compared with other existing methods. Future studies will focus on WSDR tasks under low-SNR and open-set conditions to deal with the complexities of real-world electromagnetic environments.

## Figures and Tables

**Table 1:** Summary of prior research methods: merits and limitations.

Approach	Key Merits	Identified Limitations/Research Gap
[21,22,23]	Directly estimates signal carrier frequency and bandwidth with a simple structure and high accuracy	Insufficient capability in detecting signals in dense scenarios
[24,25]	Pioneered the task definition and solution paradigm for time–frequency-domain signal localization	Lacks modulation classification capability
[26]	Constructs a time–frequency-domain framework for multi-signal detection and classification; uses eye diagrams and constellation diagrams as features to distinguish phase-modulated signals	Excessively high complexity in modulation classification; non-end-to-end model
[17,18,19,27]	Based on an anchor-free architecture, designed specifically to adapt to signal characteristics	Insufficient capability in detecting overlapping signals
[28]	Enhances the robustness of signal detection and identification in unknown channels	Inadequate ability to capture the duration of discontinuous burst signals

**Table 2:** The parameters of the WBSig53 dataset and training scheme.

Parameters	Value
Data type	Complex-valued I/Q
Sample length	262,144
SNR	[0,30] dB
Modulation class	ask, fsk, ofdm, pam, psk, qam
Nfft size	512
Window function	Blackman
Overlap	0
Image size	512, 512, 3
Training set	250,000
Validation set	25,000
Epoch	140
Batchsize	128
Learning rate	0.0001

**Table 3:** Ablation experiments on data augmentation.

None	Scaling and Cropping	Color	Flipping	Mosaic	mAP(%)
✔					84.2
	✔				86.1 **(+1.9) **
		✔			83.5 **(−0.7) **
			✔		85.3 **(+1.1) **
	✔		✔	✔	86.8 **(+2.6) **

**Table 4:** Ablation experiments on network structure.

Baseline	Basic–SPM	CA	Elliptical	FPS	Params	mAP(%)
✔				77	19.7M	86.8
✔	✔			72	25.6M	87.7 **(+0.9) **
✔	✔	✔		63	25.7M	88.0 **(+1.2) **
✔	✔	✔	✔	63	25.7M	88.2 **(+1.4) **

**Table 5:** Comparative experiments under different post-processing schemes.

Post-Processing	mAP (%)	AP_*s*_ (%)	AP_*m*_ (%)	AP_*l*_ (%)	FPS
soft−NMS	88.3	62.8	86.7	94.2	46
TFCO(sum_conf) + soft−NMS	85.3	59.4	83.6	91.8	45
TFCO(max_freq) + soft−NMS	85.1	59.6	83.2	91.6	45
TFCO(max_conf ≥ 0.6)	87.5	61.9	86.0	93.7	46
TFCO(max_conf ≥ 0.6) + soft−NMS	87.7	62.0	86.1	93.8	45
TFCO(max_conf ≥ 0.8)	88.3	62.8	86.8	94.1	46
TFCO(max_conf ≥ 0.8) + soft−NMS	88.4	62.9	86.8	94.2	45

**Table 6:** Comparative experiment on WBSig53 dataset.

Model	Backbone	mAP (%)	AP_50_ (%)	AP_75_ (%)	AP_*s*_ (%)	AP_*m*_ (%)	AP_*l*_ (%)	mAR (%)
Faster R-CNN [10]	ResNet-50-FPN	81.80	89.80	86.10	56.70	81.90	88.20	90.20
RetinaNet [11]	ResNet-50-FPN	83.40	91.60	87.90	63.60	84.10	88.60	91.70
YOLOv3 [12]	DarkNet-53	84.60	94.90	91.50	64.20	83.20	88.10	89.80
FCOS [13]	ResNet-50-FPN-DCN	82.50	89.70	86.90	61.40	83.60	84.90	91.90
Dynamic R-CNN [14]	ResNet-50-FPN	84.30	91.10	88.30	60.20	84.40	90.00	91.50
DAB-DETR [35]	ResNet-50	83.20	90.60	88.00	64.20	82.80	88.20	93.00
Conditional-DETR [36]	ResNet-50	85.50	93.60	91.00	67.40	85.50	90.40	92.80
YOLOv5-small(*)	CSPDarknet-53	58.50	72.37	64.29	38.35	56.68	54.27	64.35
PSPNet-B4(*)	EfficientNet-B4	51.84	67.23	56.54	16.85	47.59	68.53	62.61
Mask2Former-B4(*)	EfficientNet-B4	27.03	32.13	29.22	08.23	22.85	33.38	52.81
DETR-B4-Nano(*)	EfficientNet-B4	80.65	88.64	85.41	59.24	78.48	88.48	86.03
TFDP-SANet	DLA-34-SPM	88.20	95.80	93.20	62.80	86.70	94.10	92.80

**Table 7:** Comparison of model complexity and inference performance among detection methods.

Model	Params	FPS	FLOPs
Faster R-CNN [10]	41.4 M	37	63.2 G
RetinaNet [11]	36.4 M	43	52.8 G
YOLOv3 [12]	61.5 M	**51**	49.6 G
FCOS [13]	33.1 M	25	37.7 G
Dynamic R-CNN [14]	41.4 M	38	63.2 G
DAB-DETR [35]	43.7 M	21	29.0 G
Conditional-DETR [36]	43.4 M	26	28.2 G
**TFDP-SANet**	**25.7 M**	47	**26.9 G **

## Data Availability

The original data presented in the study are openly available in GitHub at https://github.com/TorchDSP/torchsig (accessed on 3 December 2025) and COCO at https://cocodataset.org (accessed on 3 December 2025).
